# Association between chronic stress-related amygdala metabolic activity and distant metastasis in colorectal cancer

**DOI:** 10.3389/fendo.2026.1747732

**Published:** 2026-02-03

**Authors:** Hyun Joo Kim, Sejin Ha, Chanmin Joung, Sungeun Kim, Kisoo Pahk

**Affiliations:** 1 Department of Nuclear Medicine, Korea University College of Medicine, Seoul, Republic of Korea; 2 Division of Nuclear Medicine, Department of Radiology, Uijeongbu St. Mary’s Hospital, College of Medicine, The Catholic University of Korea, Seoul, Republic of Korea; 3 Graduate School of Biomedical Sciences, University of Texas Southwestern Medical Center, Dallas, TX, United States

**Keywords:** amygdala, colorectal cancer, metastasis, positron emission tomography, stress

## Abstract

**Background:**

Chronic stress has been implicated in cancer progression through neuroendocrine and inflammatory pathways, but its role in colorectal cancer (CRC) remains uncertain. The amygdala, a key stress-responsive brain structure, demonstrates measurable metabolic activity on ^18^F-fluorodeoxyglucose positron emission tomography/computed tomography (^18^F-FDG PET/CT) and may serve as a surrogate imaging biomarker of chronic stress. This study aimed to investigate whether elevated amygdala metabolic activity (AmygA) is associated with distant metastasis in patients with CRC.

**Patients and methods:**

This study included patients with newly diagnosed CRC who underwent pre-treatment ¹^8^F-FDG PET/CT and curative-intent surgery between January 2019 and December 2023. AmygA was defined as the ratio of maximum standardized uptake value (SUV_max_) of the amygdala to the mean standardized uptake value (SUV_mean_) of the ipsilateral temporal lobe. Receiver-operating characteristic curve analysis determined the optimal AmygA threshold for predicting distant metastasis, and multivariable logistic regression identified independent predictors.

**Results:**

Seventy-six patients were analyzed, of whom 21 (27.6%) had distant metastasis. AmygA was significantly higher in patients with distant metastasis than in those without (1.17 ± 0.06 vs. 1.08 ± 0.06; *p* < 0.001). The optimal AmygA cutoff value for predicting distant metastasis was 1.159, yielding 71.4% sensitivity and 89.1% specificity (area under the curve = 0.844; *p* < 0.001). Univariable analysis identified advanced T stage, lymph node metastasis, elevated AmygA, increased spleen SUV_max_, and higher serum tumor marker levels as significant variables. In prespecified parsimonious multivariable logistic regression models with bootstrap internal validation, elevated AmygA (> 1.159) remained independently associated with distant metastasis.

**Conclusions:**

Elevated amygdala metabolic activity on pre-treatment ¹^8^F-FDG PET/CT, a surrogate marker of chronic stress, was independently associated with distant metastasis in CRC. AmygA might serve as a novel imaging biomarker for risk stratification and offer insight into stress-related neural mechanisms underlying metastatic progression.

## Introduction

Colorectal cancer (CRC) is the third most commonly diagnosed malignancy and a major contributor to cancer-related deaths worldwide (1, 2). Although advances in therapy have improved outcomes for localized CRC, metastatic progression remains the primary determinant of poor prognosis (1, 3–5). Emerging evidence suggests that chronic stress, resulting from prolonged exposure to persistent stressors, plays a significant role in cancer progression through multiple biological mechanisms (6–10).

Chronic stress stimulates the hypothalamic-pituitary-adrenal (HPA) axis and the sympathetic nervous system, leading to elevated release of stress hormones, including glucocorticoids and norepinephrine. These hormones facilitate tumor development and progression by causing DNA damage, suppressing p53 tumor-suppressive activity, and modifying the tumor microenvironment. Additionally, chronic stress promotes systemic inflammation by increasing circulating pro-inflammatory cells and upregulating pro-inflammatory cytokines, thus fostering a pro-tumorigenic state. Furthermore, chronic stress facilitates the formation of neutrophil extracellular traps (NETs) through glucocorticoid-mediated pathways, promotes the recruitment of myeloid-derived suppressor cells (MDSCs) via the TAM/CXCL1–CXCR2 axis, and further supports tumor progression by creating a microenvironment conducive to metastasis. Empirical evidence suggests that chronic stress is associated with cancer risk and tumor aggressiveness across multiple malignancies, including stomach (11, 12), lung (13, 14), breast (15–17), endometrium (18), head and neck (19), and skin cancers (20, 21).

The amygdalae are key components of the limbic system located in the medial temporal lobes and are essential for regulating endocrine, autonomic, and behavioral responses to psychological stress. Previous studies have demonstrated that amygdala metabolic activity (AmygA) can be quantitatively assessed using ^18^F-fluorodeoxyglucose positron emission tomography/computed tomography (^18^F-FDG PET/CT) with excellent reproducibility (18, 19, 22, 23). Increased AmygA has been reported to predict chronic stress and to be associated with cancer progression (18, 19), highlighting its potential as an imaging biomarker of stress-related oncogenic activity. These findings suggest AmygA may serve as an imaging biomarker reflecting chronic stress burden.

Despite a growing body of evidence linking chronic stress to cancer pathogenesis, its relationship with CRC remains inconclusive. A large-scale prospective cohort study conducted in 2017 failed to establish a definitive association (24), but recent findings suggest that chronic stress may contribute to colorectal cancer progression by enhancing glycolysis and inducing dysbiosis (25, 26). Therefore, we investigated whether elevated AmygA measured on ^18^F-FDG PET/CT is associated with distant metastasis in patients with CRC.

## Patients and methods

### Patients

From January 2019 to December 2023, 76 patients with newly diagnosed colorectal cancer who underwent pre-treatment ^18^F-FDG PET/CT imaging and curative-intent surgery were retrospectively analyzed.

**Figure 1:**
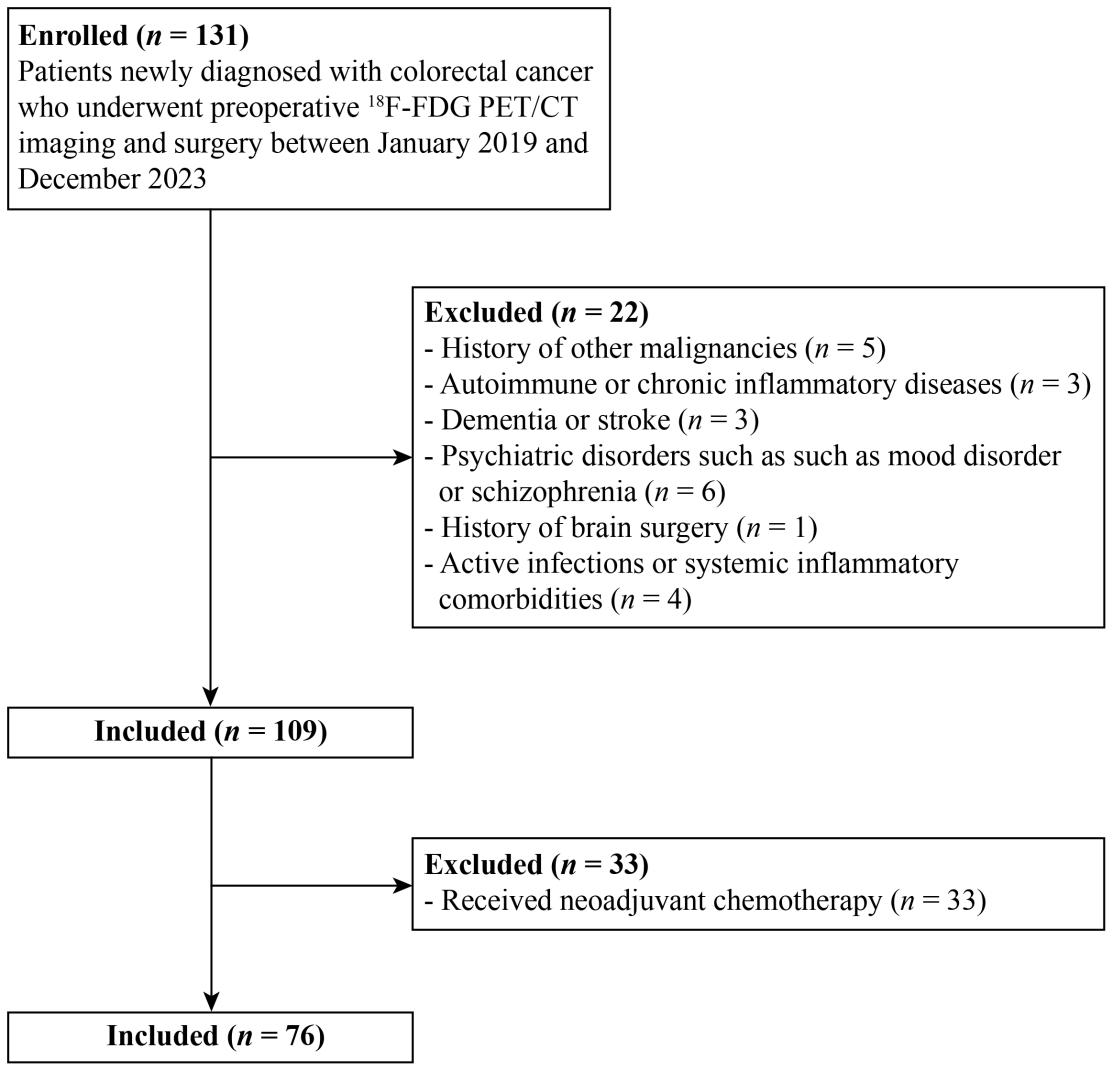
Patient flow diagram. ^18^F-FDG PET/CT; ^18^F-fluorodeoxyglucose positron emission tomography/computed tomography.

Patients with a history of other malignancies, autoimmune or chronic inflammatory diseases, neurologic disorders (dementia or stroke), or psychiatric conditions (mood or psychotic disorders) were excluded. Those with a prior history of brain surgery before ^18^F-FDG PET/CT or with active infection or systemic inflammatory comorbidities were also excluded. To minimize staging bias, 33 patients who had received chemotherapy before surgery were excluded, as their ^18^F-FDG PET/CT was performed prior to treatment whereas pathological staging was assessed after treatment, potentially leading to treatment-related downstaging. Ultimately, 76 patients met the inclusion criteria and were included in the final analysis (**Figure 1**).

This study was approved by the Institutional Review Board of Korea University Anam Hospital (Approval No. 2024AN0550). Given the retrospective nature of the study, the board waived the requirement for informed consent.

### Tumor staging

Tumor staging was performed according to the American Joint Committee on Cancer (AJCC) 8th edition (27). Pathologic T and N staging were confirmed by histopathological analysis of resected specimens following definitive surgery. M staging, indicating the presence of distant metastasis, was radiologically determined using contrast-enhanced CT of the chest, abdomen, and pelvis with supplemental MRI or ^18^F-FDG PET/CT imaging.

### Data collection, anthropometric, and laboratory assessments

Body mass index (BMI) was calculated by dividing weight in kilograms by the square of height in meters (kg/m²). Blood samples were obtained within the week before surgery. C-reactive protein (CRP) levels were measured using a chemiluminescence immunoassay (Beckman Coulter, Brea, CA, USA). Serum carcinoembryonic antigen (CEA) and carbohydrate antigen 19-9 (CA 19-9) levels were assessed by radioimmunoassay (Gamma Pro, Kaien, Seoul, Korea). Smoking status was categorized as ‘yes’ for individuals with any history of smoking and ‘no’ for never-smokers (28, 29). Alcohol consumption was classified as “yes” for individuals who consumed alcohol at least three times per week and “no” otherwise; each serving corresponded to 355 mL (12 fl oz) of beer or an equivalent amount of alcohol (28, 30).

### ^18^F-FDG PET/CT

All patients fasted overnight before undergoing ^18^F-FDG PET/CT imaging to maintain blood glucose levels below 180 mg/dL. PET/CT imaging was performed approximately 60 minutes after intravenous injection of 5.29 MBq/kg of ^18^F-FDG, covering the region from the skull vertex to the proximal thighs. A PET/CT scanner (Gemini TF, Philips Medical Systems, Cleveland, OH, USA) incorporating a lutetium–yttrium oxyorthosilicate full-ring PET detector with time-of-flight capability and a 16-slice helical CT scanner was used. The procedure began with a CT scan (120 kVp, 50 mA, 4 mm slice thickness), which was performed for attenuation correction, and was followed by a PET scan conducted with a spatial resolution of 4.4 mm and an axial field of view of 18 cm. The PET scan covered nine-bed positions, with an acquisition time of one minute per position. Images were reconstructed using a three-dimensional row-action maximum likelihood algorithm with iterative reconstruction techniques.

### Image analysis

Two board-certified nuclear medicine physicians independently reviewed ^18^F-FDG PET/CT images on a commercially available workstation (MIM Software, version 7.2.10, GE Healthcare, Cleveland, OH, USA). Both readers were blinded to all clinical and pathological information.

Quantitative analysis of ^18^F-FDG uptakes was performed using standardized uptake values (SUVs). Bilateral amygdalae were delineated on axial PET/CT images using established anatomical landmarks. The anterior boundary was defined as the inferior margin of the lateral ventricles adjacent to the thalamus, and the posterior boundary as the crux of the fornix located anterior to the basilar artery. The internal capsule defined the lateral and inferior boundaries. Circular 15-mm regions of interest (ROIs) were manually placed over each amygdala, and the maximum SUV (SUV_max_) within each ROI was recorded. The average of bilateral SUV_max_ values was designated as amygdala SUV_max_. Amygdala metabolic activity (AmygA) was defined as the ratio of amygdala SUV_max_ to the mean SUV (SUV_mean_) of the ipsilateral temporal lobe while maintaining alignment with the same plane where the amygdala ROIs were located (18, 22, 23, 31).

Elevated metabolic activity in the spleen and bone marrow on ^18^F-FDG PET/CT is a recognized surrogate marker of increased myelopoietic activity and systemic inflammation (32, 33). ROIs were drawn over the entire spleen and axial slices of vertebral bodies from L3–L5 to assess metabolic activity in spleen and bone marrow (BM). The average maximum standardized uptake values (SUV_max_) across these ROIs were calculated and recorded as spleen SUV_max_ and BM SUV_max_, respectively, in accordance with previously validated protocols (18, 22, 34). Interobserver correlation coefficients of the measured SUV indices were greater than 0.9.

### Statistical analysis

Results are presented as means ± standard deviations unless otherwise specified. Categorical variables were compared using the Chi-squared (χ²) test or Fisher’s exact test, as appropriate. The normality of continuous variables was evaluated using the Shapiro–Wilk test. The Student’s *t* test was used to analyze parametric data, and the Mann–Whitney *U* test was used for nonparametric data. Receiver-operating characteristic (ROC) curve analysis was performed to determine the optimal cutoff value of AmygA for predicting distant metastasis. Area under the curve (AUC), sensitivity, and specificity were calculated. To identify factors independently associated with distant metastasis, both univariable and multivariable logistic regression analyses were performed. Given the limited number of metastatic events, multivariable inference was based on prespecified parsimonious logistic regression models. AmygA was analyzed as a dichotomous variable using a ROC-derived cutoff. Internal validation was performed using bootstrap resampling (1,000 resamples), and bias-corrected and accelerated (BCa) 95% confidence intervals were obtained for multivariable odds ratios. Spearman’s rank correlation coefficients were used to evaluate associations between AmygA and systemic inflammatory markers. Statistical analysis was performed using MedCalc software version 23.2.1 (MedCalc Software Ltd, Ostend, Belgium) and SPSS software version 17.0 (SPSS Inc, Chicago, IL, USA). Statistical significance was accepted for *p* values < 0.05. The Bonferroni correction was applied to adjust for multiple comparisons.

## Results

### Patient characteristics

Seventy-six CRC patients of mean age 66.6 ± 13.0 years (range, 34–88 years) were included. The cohort consisted of 44 men (57.9%) and 32 women (42.1%). The mean body mass index (BMI) was 23.3 ± 3.9 kg/m² (range, 13.7–35.6 kg/m²).

Regarding primary tumor locations, the most common sites were the sigmoid colon (30 patients, 39.5%) and rectum (20 patients, 26.3%). Histologically, most tumors were moderately differentiated (67 patients, 88.2%). Based on pathologic evaluation, T3 was the most prevalent stage and was observed in 43 patients (56.6%). Regarding nodal staging, 41 patients (53.9%) exhibited lymph node metastasis, whereas 35 patients (46.1%) showed no regional lymph node involvement (N0). Distant metastasis (M1) was identified radiologically in 21 patients (27.6%), while 55 patients (72.4%) showed no evidence of distant metastasis (M0) at diagnosis. According to the AJCC 8th edition staging system, 4 patients (5.3%) were stage I, 26 patients (34.2%) were stage II, 25 patients (32.9%) were stage III, and 21 patients (27.6%) were stage IV. Detailed patient characteristics are summarized in **Table 1**.

**Table 1:** Patient characteristics.

Variables	Total patients (*n* = 76)
Age (years)	66.6 ± 13.0
Sex, *n* (%)	
Male	44 (57.9)
Female	32 (42.1)
BMI (kg/m^2^)	23.3 ± 3.9
Smoking, *n* (%)	
No	54 (71.1)
Yes	22 (28.9)
Alcohol, *n* (%)	
No	51 (67.1)
Yes	25 (32.9)
HTN, *n* (%)	
No	34 (44.7)
Yes	42 (55.3)
DM, *n* (%)	
No	61 (80.3)
Yes	15 (19.7)
Dyslipidemia, *n* (%)	
No	58 (76.3)
Yes	18 (23.7)
Primary tumor site, *n* (%)	
Appendix	2 (2.6)
Ascending colon	8 (10.5)
Transverse colon	7 (9.2)
Descending colon	2 (2.6)
Sigmoid colon	30 (39.5)
Rectosigmoid junction	7 (9.2)
Rectum	20 (26.3)
Histologic grade, *n* (%)	
1	6 (7.9)
2	67 (88.2)
3	3 (3.9)
T stage, *n* (%)	
1	3 (3.9)
2	8 (10.5)
3	43 (56.6)
4a	15 (19.7)
4b	7 (9.2)
N stage, *n* (%)	
0	35 (46.1)
1a	10 (13.2)
1b	11 (14.5)
1c	2 (2.6)
2a	12 (15.8)
2b	6 (7.9)
*M stage, *n* (%)	
0	55 (72.4)
1	21 (27.6)
AJCC stage, *n* (%)	
I	4 (5.3)
II	26 (34.2)
III	25 (32.9)
IV	21 (27.6)

Data are presented as mean ± standard deviation (SD) unless otherwise indicated.

BMI, body mass index; Alcohol, Alcohol consumption; HTN, hypertension; DM, diabetes mellitus; AJCC, American Joint Committee on Cancer; T, tumor; N, node; M, metastasis.

*Distant metastasis was defined as tumor spread to non-regional lymph nodes, distant organs, or the peritoneum, according to the AJCC 8th edition staging system.

### Comparison of clinical and laboratory characteristics by distant metastatic status

Higher T stage and lymph node metastasis were significantly associated with distant metastasis. The proportion of patients with a T4 tumor was significantly higher in the distant metastasis-positive group than in the non-distant metastasis group (52.4% vs. 20.0%, *p* = 0.006), and lymph node metastasis was also more frequent in patients with distant metastasis (76.2% vs. 45.5%, *p* = 0.02). Histologic grade showed a trend towards a higher proportion of poorly differentiated tumors in the metastasis group (9.5% vs. 1.8%), but the difference was not statistically significant (*p* = 0.13). Also, patients with distant metastasis had a significantly higher spleen SUV_max_ value than those without distant metastasis (3.1 ± 0.4 vs. 2.8 ± 0.4, *p* = 0.02), whereas no significant difference was observed for primary tumor SUV_max_ or BM SUV_max_.

Neither age, sex, BMI, comorbidities (hypertension, diabetes mellitus, or dyslipidemia), lifestyle factors (smoking or alcohol consumption), nor laboratory parameters (CRP, WBC, CEA, or CA 19-9) were significantly associated with distant metastasis. Detailed clinical and laboratory characteristics are summarized in **Table 2**. Normality test results and the corresponding test selection for continuous variables are provided in **Supplementary Table 1**.

**Table 2:** Comparison of clinical and laboratory characteristics of colorectal cancer patients with or without distant metastasis.

Variables	Patients with distant metastasis (*n* = 21)	Patients without distant metastasis (*n* = 55)	*p*
Age (years)	66.9 ± 14.3	66.5 ± 12.6	0.92
Sex, *n* (%)			0.55
Male	11 (52.4)	33 (60)	
Female	10 (47.6)	22 (40)	
BMI (kg/m^2^)	23.0 ± 4.9	23.5 ± 3.5	0.69
Smoking, *n* (%)			0.54
No	16 (76.2)	38 (69.1)	
Yes	5 (23.8)	17 (30.9)	
Alcohol, *n* (%)			0.96
No	14 (66.7)	37 (67.3)	
Yes	7 (33.3)	18 (32.7)	
HTN, *n* (%)			0.76
No	10 (47.6)	24 (43.6)	
Yes	11 (52.4)	31 (56.4)	
DM, *n* (%)			0.46
No	18 (85.7)	43 (78.2)	
Yes	3 (14.3)	12 (21.8)	
Dyslipidemia, *n* (%)			0.56
No	17 (81.0)	41 (74.5)	
Yes	4 (19.0)	12 (25.5)	
Histologic grade, *n* (%)			0.13
1, 2	19 (90.5)	54 (98.2)	
3	2 (9.5)	1 (1.8)	
T stage, *n* (%)			0.006*
T1–T3	10 (47.6)	44 (80.0)	
T4	11 (52.4)	11 (20.0)	
Lymph node metastasis, *n* (%)			0.02*
Negative	5 (23.8)	30 (54.5)	
Positive	16 (76.2)	25 (45.5)	
CRP (mg/L)	13.3 ± 18.6	9.4 ± 16.6	0.41
WBC (10^3^/μL)	8.0 ± 3.2	7.2 ± 2.9	0.30
CEA (ng/mL)	68.3 ± 165.9	13.2 ± 50.3	0.15
CA19-9 (IU/mL)	640.7 ± 2248.2	32.2 ± 78.6	0.23
Primary tumor SUV_max_	19.7 ± 10.7	17.7 ± 10.0	0.46
BM SUV_max_	2.9 ± 0.5	2.7 ± 0.5	0.10
Spleen SUV_max_	3.1 ± 0.4	2.8 ± 0.4	0.02*

Data are presented as mean ± standard deviation (SD) unless otherwise indicated.

BMI, body mass index; Alcohol, Alcohol consumption; HTN, hypertension; DM, diabetes mellitus; T, tumor; CRP, C-reactive protein; WBC, white blood cell count; CEA, carcinoembryonic antigen; CA 19-9, carbohydrate antigen 19-9; BM, bone marrow; SUV_max_, maximum standardized uptake value.

*Statistically significant.

### The independent association between AmygA and distant metastasis

**Figure 2:**
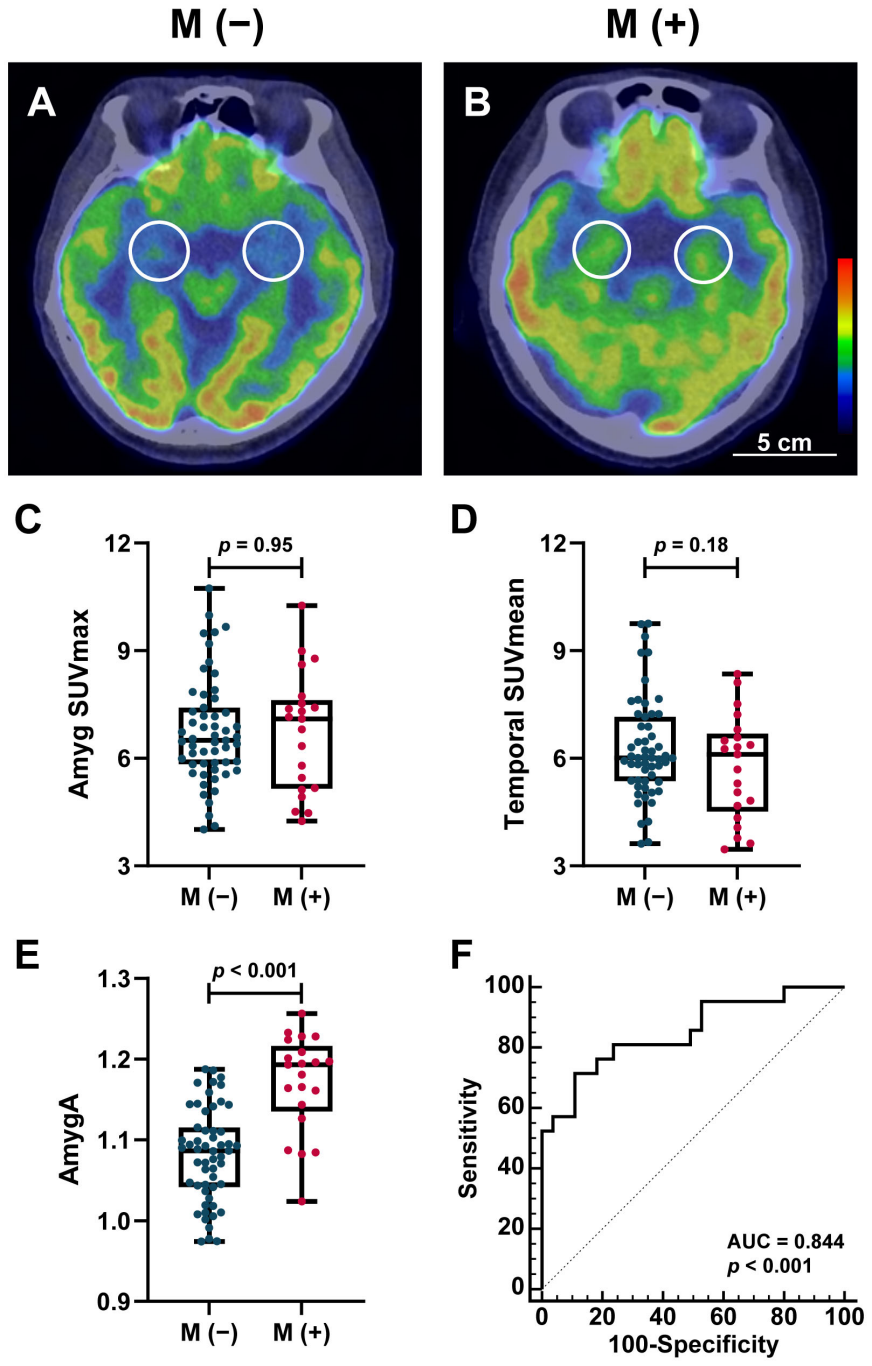
Representative axial ^18^F-FDG PET/CT images showing amygdala metabolic activity (AmygA) in patients without **(A)** and with **(B)** distant metastasis. Amygdalae are indicated by circular regions of interest. Box plot comparing Amyg SUV_max_**(C)**, Temporal SUV_mean_**(D)**, and AmygA **(E)** in patients with or without distant metastasis. Receiver operating characteristic (ROC) curve analysis of AmygA for predicting distant metastasis is shown in **(F)**. The optimal AmygA cutoff value of 1.159 was determined using the maximum Youden index, defined as [sensitivity − (1 − specificity)]. M (-), *n* = 55; M (+), *n* = 21. M (-), without distant metastasis; M (+), with distant metastasis; SUV_max_, maximum standardized uptake value; Amyg SUV_max_, SUV_max_ of amygdala; SUV_mean_, mean standardized uptake value; AUC, area under the curve.

Representative axial ^18^F-FDG PET/CT images demonstrated AmygA in patients with distant metastasis (**Figures 2A, B**), and quantitatively, AmygA values were significantly higher in patients with distant metastasis (1.17 ± 0.06 vs. 1.08 ± 0.06, *p* < 0.001; **Figure 2E**). In contrast, neither amygdala SUV_max_ (*p* = 0.95; **Figure 2C**) nor temporal lobe SUV_mean_ (*p* = 0.18; **Figure 2D**) showed a significant difference. ROC curve analysis identified an optimal AmygA cutoff value of 1.159 for predicting distant metastasis, yielding a sensitivity of 71.4% and a specificity of 89.1% with an AUC of 0.844 (*p* < 0.001) (**Figure 2F**). We additionally performed subgroup ROC analyses of AmygA for discriminating distant metastasis, stratified by primary site, tumor stage, and inflammatory status. AmygA demonstrated good predictive performance for distant metastasis across all subgroups, with no significant differences in AUCs among the subgroups. The subgroup AUCs with bootstrap BCa 95% confidence intervals are summarized in **Supplementary Table 3** and illustrated in **Supplementary Figure 1**.

Univariable logistic regression analyses were used to screen candidate variables. Multivariable inference was based on prespecified parsimonious models with bootstrap BCa 95% confidence intervals (**Table 3**). For transparency, the original full univariable screening and extended multivariable results are provided in **Supplementary Table 2**. Because T stage and lymph node status represent related measures of tumor burden, they were evaluated in separate prespecified models (Model 1 and Model 2, respectively), while spleen SUV_max_ was included as a single inflammatory surrogate covariate to limit model complexity given the limited number of metastatic events.

**Table 3:** Prespecified parsimonious multivariable logistic regression models for predicting distant metastasis.

Variables	Model 1		Model 2	
	OR (BCa 95% CI)	*p*	OR (BCa 95% CI)	*p*
AmygA (≤ 1.159 vs. > 1.159)	15.28 (1.43–782.68)	< 0.001*	14.53 (3.10–22.64)	< 0.001*
T stage (T1–T3 vs. T4)	3.48 (0.74–64.07)	0.08		
Lymph node metastasis (negative vs. positive)			2.01 (0.33–18.36)	0.32
Spleen SUV_max_ (continuous)	7.01 (1.52–929.20)	0.02	5.81 (1.30–343.78)	0.02

BCa, bias-corrected and accelerated; AmygA, amygdala metabolic activity; T, tumor; SUV_max_, maximum standardized uptake value.

Model 1 included AmygA (≤ 1.159 vs. > 1.159), T stage (T1–T3 vs. T4), and spleen SUV_max_.

Model 2 included AmygA (≤ 1.159 vs. > 1.159), lymph node metastasis (negative vs. positive), and spleen SUV_max_.

For spleen SUV_max_, odds ratios were expressed per 1-unit increase.

Bootstrap internal validation was performed using 1,000 resamples, and BCa 95% confidence intervals are presented.

*Statistically significant after Bonferroni correction for 3 comparisons (significance threshold: *p* < 0.0167).

Univariable logistic regression analysis identified several variables significantly associated with distant metastasis in CRC patients, including advanced primary tumor stage (T4 vs. T1–T3; OR = 4.40, 95% CI: 1.49–12.98, *p* = 0.007), the presence of lymph node metastasis (OR = 5.38, 95% CI: 1.42–20.38, *p* = 0.005), elevated AmygA (> 1.159; OR = 14.40, 95% CI: 4.27–48.57, p < 0.001), increased spleen SUV_max_ (OR = 3.92, 95% CI: 1.18–13.08, *p* = 0.02), and higher serum levels of CEA (per ng/mL; OR = 1.01, 95% CI: 1.00–1.02, *p* = 0.04) and CA 19-9 (per U/mL; OR = 1.01, 95% CI: 1.00–1.01, *p* = 0.002) (**Supplementary Table 2**).

The prespecified parsimonious multivariable logistic regression models incorporating bootstrap BCa 95% confidence intervals showed that AmygA, dichotomized using the ROC-derived cutoff, remained independently associated with distant metastasis and was the only variable that met the Bonferroni-corrected significance threshold (Model 1: OR = 15.28, BCa 95% CI: 1.43–782.68, *p* < 0.001; Model 2: OR = 14.53, BCa 95% CI: 3.10–22.64, *p* < 0.001) after adjustment for tumor burden (T stage or lymph node status) and an inflammatory surrogate marker (spleen SUV_max_) (**Table 3**).

### Associations between AmygA and systemic inflammatory markers

No significant correlations were found between AmygA and systemic inflammatory markers, including BM SUV_max_ (*r* = 0.18, *p* = 0.13), spleen SUV_max_ (*r* = 0.06, *p* = 0.62), CRP (*r* = 0.13, *p* = 0.30), and WBC (*r* = −0.03, *p* = 0.81) (**Table 4**).

**Table 4:** Spearman’s correlation coefficients for the relationship between AmygA and systemic inflammatory markers.

Variables	*r*	*p*
BM SUV_max_	0.18	0.13
Spleen SUV_max_	0.06	0.62
CRP	0.13	0.30
WBC	−0.03	0.81

AmygA, amygdala metabolic activity; BM, bone marrow; SUV_max_, maximum standardized uptake value; CRP, C-reactive protein; WBC, white blood cell count.

## Discussion

This study indicates for the first time that chronic stress, as reflected by elevated AmygA, is independently associated with the presence of distant metastasis in CRC patients. Notably, AmygA retained its independent association with distant metastasis even after adjusting for established prognostic factors, including advanced T stage and regional lymph node involvement. The significantly higher AmygA observed in patients with metastatic disease suggests that chronic stress contributes meaningfully to the progression of metastasis.

Our findings are consistent with prior studies that have reported that elevated AmygA measured by ^18^F-FDG PET/CT serves as an imaging-based biomarker of chronic stress burden (18, 19, 22, 23, 35). Moreover, previous studies have reported significant associations between AmygA and adverse oncologic outcomes in malignancies, such as mortality and cancer progression in head and neck and endometrial cancers (18, 19). Our results extend these observations to CRC by indicating that elevated AmygA is associated with CRC progression and metastatic potential.

Published AmygA distributions differ across disease populations. In an osteoporosis cohort, AmygA was 0.81 ± 0.16 in patients with osteoporosis and 0.61 ± 0.13 in those without osteoporosis (22). In endometrial cancer, postmenopausal patients with versus without lymph node metastasis showed AmygA values of 0.93 ± 0.08 versus 0.86 ± 0.06. In contrast, no difference was observed in the premenopausal subgroup, with AmygA values of 0.89 ± 0.07 versus 0.89 ± 0.05 (18). In our CRC cohort, AmygA was 1.17 ± 0.06 in patients with distant metastasis and 1.08 ± 0.06 in those without distant metastasis, which is numerically higher than the values reported in these prior cohorts. However, direct cross-study comparison is not appropriate because AmygA is a ratio metric and may reflect differences in both amygdala uptake and reference-region metabolism, as well as cohort composition and imaging-related factors. Consistent with this heterogeneity, other studies also defined “high” amygdala activity using distribution-based thresholds rather than a single universal numeric cutoff (19, 23). Accordingly, we interpret our cutoff of 1.159 as a cohort-specific threshold intended for interpretability rather than a universal value.

**Figure 3:**
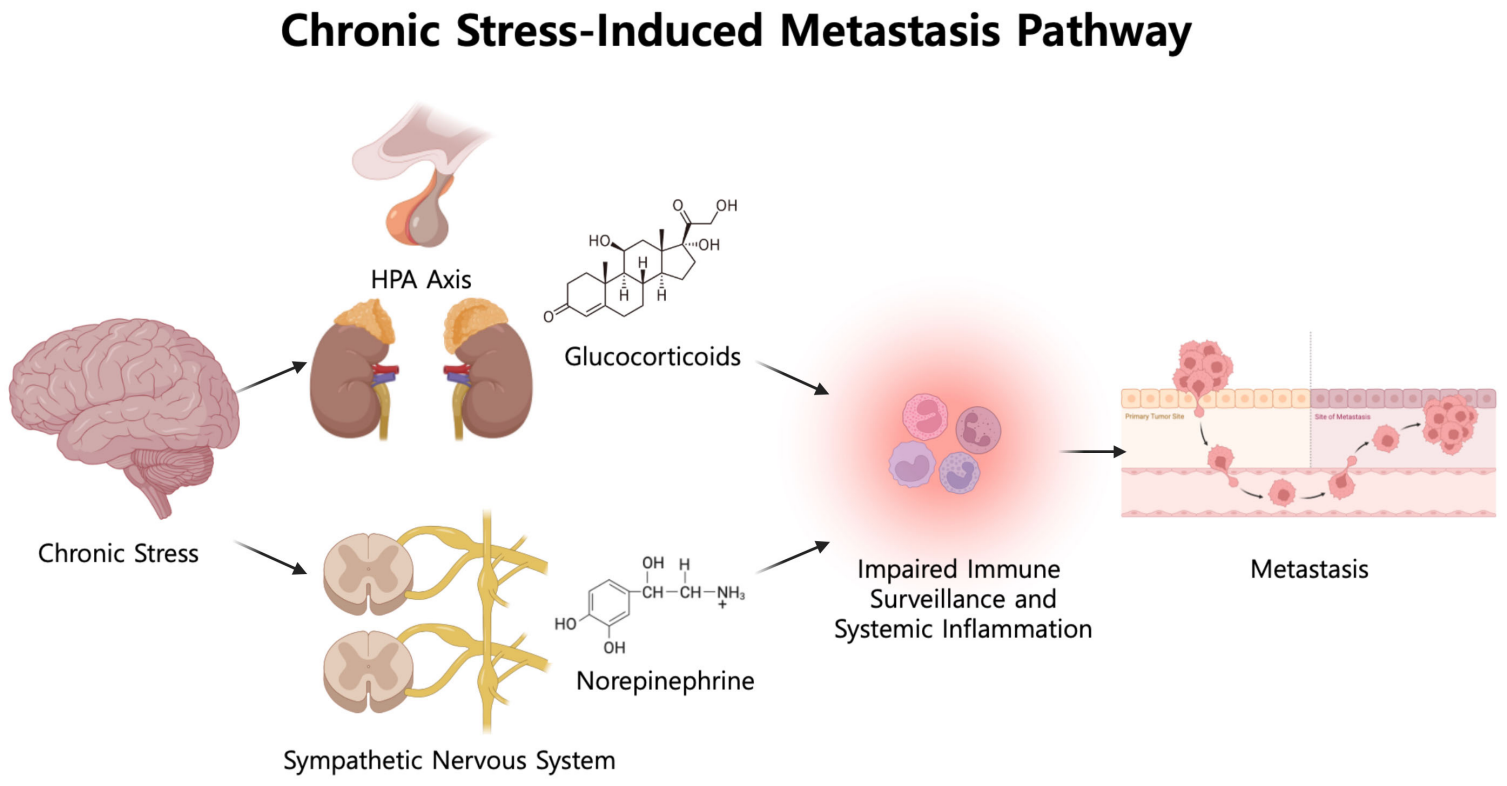
Proposed schematic linking chronic stress to elevated AmygA and metastatic potential. Chronic stress is reflected by elevated AmygA, an ^18^F-FDG PET/CT–derived imaging marker of stress-related neural metabolic activity. Chronic stress activates the HPA (hypothalamic–pituitary–adrenal) axis and the sympathetic nervous system, resulting in increased glucocorticoids and catecholamines such as norepinephrine. These neuroendocrine signals may impair anti-tumor immune surveillance and promote systemic and tumor-associated inflammatory remodeling, thereby facilitating a pro-metastatic environment.

The mechanisms underlying the association between elevated AmygA and distant metastasis in CRC remain incompletely understood. Previous studies have suggested that chronic stress may influence cancer progression by upregulating systemic inflammation (6, 7, 18, 19). Furthermore, the pro-tumorigenic effects of an inflammatory microenvironment in CRC have been well established (36). The following cascade of stress-induced inflammatory upregulation represents a biologically plausible mechanism linking chronic stress to metastatic progression in CRC (6, 7, 37–40). Chronic stress stimulates both the HPA axis and the sympathetic nervous system, resulting in elevated secretion of stress-associated hormones, including norepinephrine and glucocorticoids. These neuroendocrine changes subsequently inhibit cell-mediated immune responses and enhance systemic inflammatory activity. In addition, stress-activated sympathetic axons innervating the bone marrow stimulate hematopoietic stem cell proliferation and mobilize inflammatory monocytes, which release proinflammatory cytokines that impair immune surveillance and promote tumor invasion and distant metastasis. A schematic overview of the proposed pathway is shown in **Figure 3**.

In this study, spleen SUV_max_, a well-established surrogate marker of systemic inflammation (41–44), was elevated in CRC patients with distant metastasis and significantly predicted metastatic disease by univariable analysis. To address potential confounding by systemic inflammation, spleen SUV_max_ was included as an inflammatory surrogate covariate in the prespecified multivariable models, and the association between AmygA and distant metastasis remained significant after this adjustment. Although AmygA was positively correlated with surrogate markers of systemic inflammation, including BM SUV_max_, spleen SUV_max_, and CRP, these correlations were not significant. Several studies have reported that AmygA is significantly correlated with systemic inflammation (18, 19, 22, 23). In a cardiovascular cohort (*n* = 293), AmygA showed significant correlations with spleen SUV_max_ (*r* = 0.47, *p* < 0.001), BM SUV_max_ (*r* = 0.40, *p* < 0.001), and CRP (*r* = 0.83, *p* = 0.02) (23), and in patients with endometrial cancer (*n* = 161) and head and neck cancer (*n* = 240), AmygA was significantly correlated with CRP and BM SUV_max_ (18, 19). Furthermore, in a health-screening cohort (*n* = 115), AmygA was significantly correlated with CRP (22). This discrepancy could be attributed to the smaller sample size in our study (*n* = 76) compared with previous studies (*n* = 115–293) (18, 19, 22, 23), which may have limited our ability to detect modest associations. Accordingly, our data are insufficient to draw definitive conclusions regarding the association between AmygA and systemic inflammation in CRC.

Non-pharmacologic strategies, such as yoga, exercise, and meditation, and pharmacologic approaches, such as β-adrenergic blockade, have shown promise for mitigating stress-induced biological changes (35, 45–48), and a recent meta-analysis showed that exercise might reduce CRC risk by 13–16% (49). Furthermore, a prospective randomized clinical trial demonstrated that exercise exerts promising effects on survival among stage II and III CRC survivors (50). Interestingly, we previously found that AmygA may reflect the beneficial effects of exercise on stress-related neurobiology (35). Thus, given its ability to assess stress-related neural activity noninvasively, AmygA may have potential as an imaging-based biomarker of the efficacy of stress-reduction interventions in CRC patients.

This study has several limitations. The modest sample size and single-center, retrospective design may limit the generalizability of the findings, which should be confirmed by larger, multicenter prospective studies. Although an association between elevated AmygA and distant metastasis in CRC was observed, causality could not be inferred because of the study’s observational nature. Moreover, given the limited number of metastatic events, effect estimates showed wide confidence intervals, warranting cautious interpretation and confirmation in larger cohorts. Additionally, one patient exhibited an extremely low BMI consistent with cachexia and markedly poor general status. Such extreme outliers may disproportionately influence estimates in small cohorts. Residual confounding is possible because medication use that may influence stress-related neural activity or tumor biology (e.g., β-blockers, systemic steroids, or antidepressants) was not systematically captured and direct HPA-axis or sympathetic nervous system biomarkers were not assessed. In addition, the limited spatial resolution of current ^18^F-FDG PET/CT systems hinders detailed assessment of other stress-related brain regions, such as the hippocampus and prefrontal cortex.

## Conclusion

This study shows that chronic stress-related metabolic activity in the amygdala is closely linked to the development of distant metastases in patients with CRC. While the underlying mechanisms remain unclear, these findings offer insight into the connection between stress-related neural activity and cancer progression and highlight the potential of AmygA as a novel imaging-based biomarker of CRC aggressiveness.

## Data Availability

The original contributions presented in the study are included in the article/**Supplementary Material**. Further inquiries can be directed to the corresponding author/s.

## Supplementary material

Supplementary Figure 1 Subgroup receiver-operating characteristic (ROC) curves of AmygA for discriminating distant metastasis, stratified by primary site, tumor stage, and inflammatory status. Receiver-operating characteristic (ROC) curves of AmygA for discriminating distant metastasis stratified by **(A)** primary tumor site (colon vs. rectum), **(B)** tumor stage (T1–3 vs. T4), and **(C)** inflammatory status (low vs. high spleen SUV_max_). Spleen SUV_max_ was dichotomized at 2.9 (cohort mean).

Supplementary Table 1 Assessment of normality for continuous variables. BMI, body mass index; CRP, C-reactive protein; WBC, white blood cell count; CEA, carcinoembryonic antigen; CA 19-9, carbohydrate antigen 19-9; BM, bone marrow; SUV_max_, maximum standardized uptake value; Amyg, amygdala.

Supplementary Table 2 Univariable and multivariable analysis results for the prediction of distant metastasis OR, odds ratio; CI, confidence interval; BMI, body mass index; HTN, hypertension; DM, diabetes mellitus; CRP, C-reactive protein; WBC, white blood cell count; T, tumor; Amyg, amygdala; AmygA, amygdala metabolic activity; SUV_max_, maximum standardized uptake value; BM, bone marrow; CEA, carcinoembryonic antigen; CA 19-9, carbohydrate antigen 19-9. ORs for continuous variables are expressed per 1-unit increase. *Statistically significant. **Statistically significant after Bonferroni correction for 6 comparisons (significance threshold: *p* < 0.0083).

Supplementary Table 3 Subgroup analyses of the discriminatory performance of AmygA for distant metastasis, stratified by primary site, tumor stage, and inflammatory status AmygA, amygdala metabolic activity; AUC, area under the curve; BCa 95% CI, bias-corrected and accelerated 95% confidence interval; T, tumor; SUV_max_, maximum standardized uptake value. Comparison *p* values indicate between-subgroup differences in AUCs. Spleen SUV_max_ was categorized into high and low by 2.9, which was the mean of spleen SUV_max_ in this study.
