# Supplementary figures and images for: Association between chronic stress-related amygdala metabolic activity and distant metastasis in colorectal cancer
Source: Front Endocrinol (Lausanne). 2026 Feb 3;17:1747732. doi: 10.3389/fendo.2026.1747732 (PMC12909207; doi:10.3389/fendo.2026.1747732)

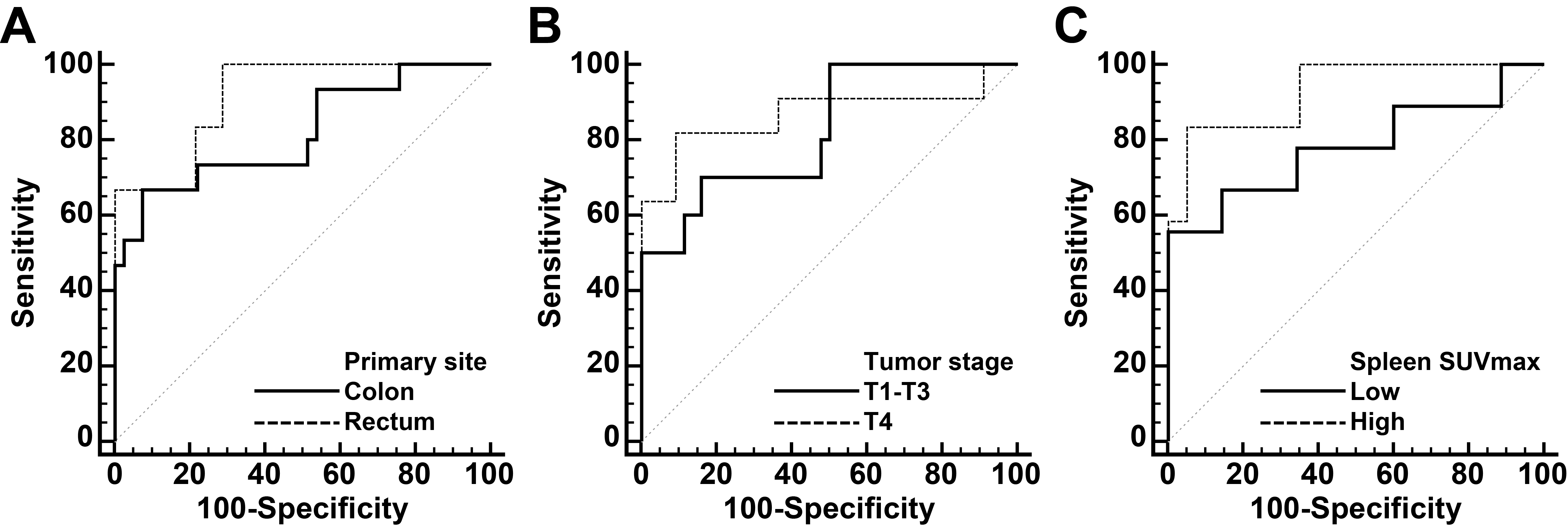

Supplement: Supplementary Figure 1 — Subgroup receiver-operating characteristic (ROC) curves of AmygA for discriminating distant metastasis, stratified by primary site, tumor stage, and inflammatory status. Receiver-operating characteristic (ROC) curves of AmygA for discriminating distant metastasis stratified by (A) primary tumor site (colon vs. rectum), (B) tumor stage (T1–3 vs. T4), and (C) inflammatory status (low vs. high spleen SUVmax). Spleen SUVmax was dichotomized at 2.9 (cohort mean). [file Image1.tif]
