# Supplementary material for: Association between chronic stress-related amygdala metabolic activity and distant metastasis in colorectal cancer
Source: Front Endocrinol (Lausanne). 2026 Feb 3;17:1747732. doi: 10.3389/fendo.2026.1747732 (PMC12909207; doi:10.3389/fendo.2026.1747732)
Supplement: Supplementary Table 1 — Assessment of normality for continuous variables. BMI, body mass index; CRP, C-reactive protein; WBC, white blood cell count; CEA, carcinoembryonic antigen; CA 19-9, carbohydrate antigen 19-9; BM, bone marrow; SUVmax, maximum standardized uptake value; Amyg, amygdala. [file Table1.docx]

Supplementary Table 1. Assessment of normality for continuous variables.

| Variables | Shapiro–Wilk test | *p* value | Normality assumption (*p* ≥ 0.05) | Test used for group comparison |
| --- | --- | --- | --- | --- |
| Age (years) | 0.966 | 0.04 | Reject | Mann–Whitney *U* test |
| BMI (kg/m^2^) | 0.990 | 0.84 | Accept | Student’s *t* test |
| CRP (mg/L) | 0.621 | < 0.001 | Reject | Mann–Whitney *U* test |
| WBC (10^3^/μL) | 0.873 | < 0.001 | Reject | Mann–Whitney *U* test |
| CEA (ng/mL) | 0.286 | < 0.001 | Reject | Mann–Whitney *U* test |
| CA19-9 (IU/mL) | 0.145 | < 0.001 | Reject | Mann–Whitney *U* test |
| Primary tumor SUV_max_ | 0.820 | < 0.001 | Reject | Mann–Whitney *U* test |
| BM SUV_max_ | 0.974 | 0.12 | Accept | Student’s *t* test |
| Spleen SUV_max_ | 0.972 | 0.08 | Accept | Student’s *t* test |
| Amyg SUV_max_ | 0.973 | 0.11 | Accept | Student’s *t* test |

BMI, body mass index; CRP, C-reactive protein; WBC, white blood cell count; CEA, carcinoembryonic antigen; CA 19-9, carbohydrate antigen 19-9; BM, bone marrow; SUV_max_, maximum standardized uptake value; Amyg, amygdala.
