# Supplementary material for: Association between chronic stress-related amygdala metabolic activity and distant metastasis in colorectal cancer
Source: Front Endocrinol (Lausanne). 2026 Feb 3;17:1747732. doi: 10.3389/fendo.2026.1747732 (PMC12909207; doi:10.3389/fendo.2026.1747732)
Supplement: Supplementary Table 2 — Univariable and multivariable analysis results for the prediction of distant metastasis OR, odds ratio; CI, confidence interval; BMI, body mass index; HTN, hypertension; DM, diabetes mellitus; CRP, C-reactive protein; WBC, white blood cell count; T, tumor; Amyg, amygdala; AmygA, amygdala metabolic activity; SUVmax, maximum standardized uptake value; BM, bone marrow; CEA, carcinoembryonic antigen; CA 19-9, carbohydrate antigen 19-9. ORs for continuous variables are expressed per 1-unit increase. *Statistically significant. **Statistically significant after Bonferroni correction for 6 comparisons (significance threshold: p < 0.0083). [file Table2.docx]

Supplementary Table 2. Univariable and multivariable analysis results for the prediction of distant metastasis

| Variables | Univariable | |  | Multivariable | |
| --- | --- | --- | --- | --- | --- |
|  | OR (95% CI) | *p* |  | OR (95% CI) | *p* |
| Age (continuous) | 1.00 (0.96–1.04) | 0.92 |  |  |  |
| Sex (male vs. female) | 1.36 (0.50–3.75) | 0.55 |  |  |  |
| BMI (continuous) | 0.97 (0.85–1.10) | 0.64 |  |  |  |
| Smoking (no vs. yes) | 0.70 (0.22–2.22) | 0.54 |  |  |  |
| Alcohol consumption (no vs. yes) | 1.03 (0.35–2.99) | 0.96 |  |  |  |
| HTN (no vs. yes) | 0.85 (0.31–2.34) | 0.76 |  |  |  |
| DM (no vs. yes) | 0.60 (0.15–2.37) | 0.45 |  |  |  |
| Dyslipidemia (no vs. yes) | 0.69 (0.14–3.40) | 0.64 |  |  |  |
| CRP (continuous) | 1.01 (0.98–1.04) | 0.42 |  |  |  |
| WBC (continuous) | 1.09 (0.93–1.28) | 0.31 |  |  |  |
| Histologic grade (1 and 2 vs. 3) | 5.68 (0.49–66.32) | 0.17 |  |  |  |
| Primary tumor SUV_max_ | 1.02 (0.97–1.07) | 0.46 |  |  |  |
| T stage (T1–T3 vs. T4) | 4.40 (1.49–12.98) | 0.007* |  | 1.73 (0.34–8.76) | 0.51 |
| Lymph node metastasis (negative vs. positive) | 5.38 (1.42–20.38) | 0.005* |  | 1.71 (0.20–14.94) | 0.63 |
| Amyg SUV_max_ (continuous) | 0.99 (0.71–1.38) | 0.95 |  |  |  |
| AmygA (≤ 1.159 vs. > 1.159) | 14.40 (4.27–48.57) | < 0.001* |  | 15.47 (2.36–101.63) | 0.004** |
| BM SUV_max_ (continuous) | 2.39 (0.83–6.91) | 0.10 |  |  |  |
| Spleen SUV_max_ (continuous) | 3.92 (1.18–13.08) | 0.02* |  | 7.69 (1.25–47.19) | 0.03 |
| CEA (continuous) | 1.01 (1.00–1.02) | 0.04* |  | 1.00 (1.00–1.01) | 0.57 |
| CA 19-9 (continuous) | 1.01 (1.00–1.01) | 0.002* |  | 1.00 (1.00–1.01) | 0.66 |

OR, odds ratio; CI, confidence interval; BMI, body mass index; HTN, hypertension; DM, diabetes mellitus; CRP, C-reactive protein; WBC, white blood cell count; T, tumor; Amyg, amygdala; AmygA, amygdala metabolic activity; SUV_max_, maximum standardized uptake value; BM, bone marrow; CEA, carcinoembryonic antigen; CA 19-9, carbohydrate antigen 19-9.

ORs for continuous variables are expressed per 1-unit increase.

*Statistically significant.

**Statistically significant after Bonferroni correction for 6 comparisons (significance threshold: *p* < 0.0083).
