# Supplementary material for: Association between chronic stress-related amygdala metabolic activity and distant metastasis in colorectal cancer
Source: Front Endocrinol (Lausanne). 2026 Feb 3;17:1747732. doi: 10.3389/fendo.2026.1747732 (PMC12909207; doi:10.3389/fendo.2026.1747732)
Supplement: Supplementary Table 3 — Subgroup analyses of the discriminatory performance of AmygA for distant metastasis, stratified by primary site, tumor stage, and inflammatory status AmygA, amygdala metabolic activity; AUC, area under the curve; BCa 95% CI, bias-corrected and accelerated 95% confidence interval; T, tumor; SUVmax, maximum standardized uptake value. Comparison p values indicate between-subgroup differences in AUCs. Spleen SUVmax was categorized into high and low by 2.9, which was the mean of spleen SUVmax in this study. [file Table3.docx]

Supplementary Table 3. Subgroup analyses of the discriminatory performance of AmygA for distant metastasis, stratified by primary site, tumor stage, and inflammatory status

| Subgroup | Variables | AUC | BCa 95% CI | *p* value | Comparison *p* value |
| --- | --- | --- | --- | --- | --- |
| Primary site | Colon | 0.82 | 0.65–0.93 | < 0.001 | 0.31 |
|  | Rectum | 0.92 | 0.70–1.00 | < 0.001 |  |
| Tumor stage | T1–3 | 0.83 | 0.62–0.94 | < 0.001 | 0.72 |
|  | T4 | 0.87 | 0.56–0.98 | < 0.001 |  |
| Inflammatory status | Low spleen SUV_max_ | 0.78 | 0.49–0.94 | 0.01 | 0.22 |
|  | High spleen SUV_max_ | 0.93 | 0.77–0.99 | < 0.001 |  |

AUC, area under the curve; BCa 95% CI, bias-corrected and accelerated 95% confidence interval; T, tumor; SUV_max_, maximum standardized uptake value.

Comparison *p* values indicate between-subgroup differences in AUCs.

Spleen SUV_max_ was categorized into high and low by 2.9, which was the mean of spleen SUV_max_ in this study.
